# *Sh3pxd2b* Mice Are a Model for Craniofacial Dysmorphology and Otitis Media

**DOI:** 10.1371/journal.pone.0022622

**Published:** 2011-07-27

**Authors:** Bin Yang, Cong Tian, Zhi-guang Zhang, Feng-chan Han, Rami Azem, Heping Yu, Ye Zheng, Ge Jin, James E. Arnold, Qing Y. Zheng

**Affiliations:** 1 Department of Oral and Maxillofacial Surgery, Guanghua School of Stomatology, Sun Yat-Sen University, Guangzhou, China; 2 Department of Otolaryngology - Head and Neck Surgery, School of Medicine, Case Western Reserve University, Cleveland, Ohio, United States of America; 3 School of Dental Medicine, Case Western Reserve University, Cleveland, Ohio, United States of America; Texas A&M University, United States of America

## Abstract

Craniofacial defects that occur through gene mutation during development increase vulnerability to eustachian tube dysfunction. These defects can lead to an increased incidence of otitis media. We examined the effects of a mutation in the *Sh3pxd2b* gene (*Sh3pxd2b^nee^*) on the progression of otitis media and hearing impairment at various developmental stages. We found that all mice that had the *Sh3pxd2b^nee^* mutation went on to develop craniofacial dysmorphologies and subsequently otitis media, by as early as 11 days of age. We found noteworthy changes in cilia and goblet cells of the middle ear mucosa in *Sh3pxd2b^nee^* mutant mice using scanning electronic microscopy. By measuring craniofacial dimensions, we determined for the first time in an animal model that this mouse has altered eustachian tube morphology consistent with a more horizontal position of the eustachian tube. All mutants were found to have hearing impairment. Expression of TNF-α and TLR2, which correlates with inflammation in otitis media, was up-regulated in the ears of mutant mice when examined by immunohistochemistry and semi-quantitative RT-PCR. The mouse model with a mutation in the *Sh3pxd2b* gene (*Sh3pxd2b^nee^*) mirrors craniofacial dysmorphology and otitis media in humans.

## Introduction

Otitis media (OM) is a prevalent problem in children under the age of 15 [1], [2]. OM can be acute or chronic and can be accompanied by excessive middle ear effusion, an accumulation of fluid in the middle ear [1], [2], [3]. By the age of 10, about 80% of children have had at least one case of OM with effusion [3], [4], [5], [6], caused by eustachian tube dysfunction. This is the leading cause of impaired hearing in children [7]. The eustachian tube is the only ventilation pathway of the middle ear cavity, interconnecting the nasopharynx and the external environment. Thus, it is crucial in clearing the middle ear of excessive effusion and in balancing middle ear pressure [8], [9], [10]. Malfunction or malformation of the bones and/or muscles around the eustachian tube can cause obstruction or a patulous condition, which may result in excessive middle ear fluid accumulation or retrograde infection through the eustachian tube [11], [12], [13]. All evidence indicates a crucial role for the eustachian tube in OM pathogenesis.

Craniofacial development influences eustachian tube function. Craniofacial dysmorphology may change the position or angle of the eustachian tube, and/or the location of surrounding muscles that control the eustachian tube. These changes can cause eustachian tube obstruction or incorrect opening and, ultimately, OM [14], [15], [16], [17], [18], [19]. Cleft palate malformation has been clearly associated with OM [20], [21]. Patients affected by craniofacial dysmorphology, but not by a cleft palate, show increased incidence of OM [15], [22]. Thus, although these conditions frequently occur together, one is sufficient for predisposition to OM.

The expression of mouse protein SH3PXD2B (SH3 and PX domains 2B, MGI: 2442062; synonymous with human TKS4) has recently been correlated with formation of podosomes and invadopodia, which mediate interactions between cells (normal and cancerous, respectively) and the extracellular matrix [23]. The SH3PXD2B protein is a member of the src tyrosine kinase substrate family. SH3PXD2B can recruit the membrane type-1 matrix metalloproteinase (MT1-MMP), resulting in extracellular matrix degeneration [23], [24]. Extracellular matrix remodeling is a significant part of normal postnatal development of several tissues and also in cancer cell invasion and migration [25], [26], [27]. The *Sh3pxd2b^nee^* mutation spontaneously arose in the mouse strain B10.A-H2^h4^/(4R)SgDVEg that was bred in the Jackson Laboratory (Bar Harbor, ME, USA). This mutant bears a single base-pair deletion in the last exon of the *Sh3pxd2b* gene on chromosome 11, resulting in a frameshift mutation and premature stop codon, thus eliminating the third and fourth SH3 domains of the SH3PXD2B protein [23], [24], [28]. *Sh3pxd2b^nee^* causes a craniofacial dysmorphology phenotype featuring a domed skull, shortened maxilla, shortened nose, glaucoma, growth retardation, adipose defects, and hearing impairment, all reported previously [24], [28]. These craniofacial defects can lead to OM, among other defects. Humans with Frank-ter Haar syndrome have a *Sh3pxd2b* gene defect that results in craniofacial dysmorphology, skeletal dysplasia, and eye and cardiac abnormalities, closely resembling the mouse phenotype [28].

Here, we show that the *Sh3pxd2b^nee^* mutation leads to progressive OM in mice through defects in craniofacial structure. *Sh3pxd2b^nee^* mutant mice exhibited a specific craniofacial dysmorphology and eustachian tube alteration and all mutants developed OM with onset as early as 11 days after birth. Mutant mice showed progressive hearing impairment, tympanic membrane retraction, and upregulated TNF-α and TLR2 in the middle ear. This evidence demonstrates the similarities in development and progression of OM between mice and humans affected by craniofacial dysmorphology.

## Materials and Methods

### Mouse husbandry and genotype

*Sh3pxd2d^nee^* heterozygous mice were obtained from the Jackson Laboratory (Bar Harbor, ME) and bred at the Wolstein Animal Research Facility at Case Western Reserve University. Because of previously reported infertility in homozygous mutants [24], the strain was maintained by heterozygous cross-mating. 52 homozygous *Sh3pxd2b^nee^* mutant mice and 66 wild-type littermate mice, from 6 days to 3 months of age, were used. All mice were genotyped by PCR and *Rsa*I restriction enzyme (New England Biolabs) digestion methods as reported previously [24]. Experimental procedures were reviewed and approved by the Health Sciences IACUC of Case Western Reserve University. (Protocol numbers: 2008-0174 and 2008-0156).

### Gross craniofacial phenotype and macroscopic ear observations

Lateral view photos of 2.5-month-old *Sh3pxd2b^nee^* mutant mice (n = 4) and wild-type littermate control mice (n = 4) were taken by digital camera (Canon 450D, Japan). Observation and comparison of the features of this mutant were made directly from the photos. Male *Sh3pxd2b^nee^* mutant mice and male wild-type littermate control mice were sacrificed by CO_2_ asphyxiation at a given time point after birth (day 6, day 11, day 14, day 16, day 21, n = 4 each phenotype at each time point); then mice were skinned and observed under a macroscope (Leica S6D, Germany). Ear and tympanic membrane photos were taken for comparison.

### Histological preparation of middle and inner ears

Bullae (including both middle and inner ear) were isolated from ears of control (n = 4 mice) and *Sh3pxd2b^nee^* (n = 3) mice at various time points (ages 6, 11, 16 and 21 days, and 2.5 months; 4 mice per age-group) for gross and histological pathology examination. After dissection, bullae were fixed in 4% PFA for 24 hours and then decalcified in 10% EDTA. For 11- to 21-day-old mice, samples were decalcified for 7 days before embedding in paraffin for sectioning. For 6- to 15-month-old mice, decalcification was for 21 days. Then samples were serially sectioned at 7 µm thickness and mounted onto slides.

### Hematoxylin and eosin staining; Mayer's mucicarmine staining

For hematoxylin and eosin (H&E) staining, a standard protocol from Rosen's lab was used (http://www.bcm.edu/rosenlab). Mayer's mucicarmine staining was used to identify goblet cells in the middle ear mucosa following the manufacturer's protocol (Electron Microscopy Sciences). Sections were examined under a light microscope (Leica DFC500, Germany). Images were acquired at 5 to 63× final magnification.

### Scanning electronic microscopy

Bullae were isolated from skulls of 21-day-old control and *Sh3pxd2b^nee^* mice (n = 4 mice, each genotype). Samples were placed in 2.5% gluteraldehyde in cacodylic acid in 0.1 M phosphate buffer (pH = 7.2) for 36 hours. After decalcification in 10% EDTA for 7 days, middle ear cavities were dissected from the bullae and then dehydrated in serial solutions of ethanol (60%, 70%, 80%, 95%, 100%) for 15 minutes each, with a second soak in 100% ethanol. Each middle ear cavity was subjected to carbon dioxide critical point drying, sputter-coated with 60/40 gold-palladium, and then viewed under a high-resolution scanning electron microscope (Hitachi S-4500, Japan).

### Scoring system for pathology of middle and inner ears

A scoring system of −/+/++/+++ was used to assess the severity of pathology in middle and inner ears. Symbols were assigned as follows: −, absence of pathology; +, very scarce pathology in the middle or inner ear; ++, pathology prevalent, but not spanning the entire middle or inner ear; +++, pathology spanning the entire middle or inner ear. The scored pathologies included middle ear effusion, inflammatory cell infiltration, tissue proliferation, goblet cell density, and inner ear effusion. A chi-square test was used to evaluate the semi-quantitative data. This method has been reported in otitis media studies [29], [30].

### Skull preparation and craniofacial measurement

To measure craniofacial dimensions, skulls were dissected and removed from mutant (n = 8) and control (n = 9) mice at age 3 months. The skulls were macerated in 1% KOH overnight to remove soft tissue and then stained with alizarin red, as previously described by M.C. Green [31]. To measure skull dimensions, a hand-held digital caliper (General Tools & Instruments) of 0.01 mm resolution was used. This method was previously validated for accuracy and precision [24], [32]. 44 landmarks were adapted from Dr. Joan Richtsmeier's publication [32], and from the standard measurement protocol in the craniofacial mutant source (Jackson Laboratory, http://craniofacial.jax.org/standard_protocols.html). The angle between the midline of the skull base and the bony part of the eustachian tube was measured for mutants and controls. Skulls were examined under an anatomical microscope and the skull base was photographed. Screen ruler software MB-Ruler 4.0 (Markus Bader, http://www.markus-bader.de/MB-Ruler/download.htm) was used to precisely locate the lines for angle measurement and to perform measurements. Measurement data were analyzed using the Student *t* test.

### Time-course ABR thresholds

Auditory-evoked brain stem response (ABR) was used to evaluate hearing in *Sh3pxd2b^nee^* mutant mice. The method has been reported previously [33]. Mice were anesthetized with avertin (0.5 mg/g body mass). A computer-aided evoked potential system (Intelligent Hearing Systems) was used to record ABR thresholds for *Sh3pxd2b^nee^* mutant and control mice from 1 to 6 months of age. Student *t* test was used to analyze the mean ABR thresholds at each time point.

### Semi-quantitative RT-PCR

RNA was isolated from the bullae of four mutant and four control 21-day-old mice using a pure-Link TM Micro-to-Midi Total RNA Purification System (Invitrogen). The SuperScript III First-Strand Synthesis System for RT-PCR was used to synthesize cDNA. Relative mRNA expression levels for *Tnf-α* and *Tlr2* were determined by by PCR using *Gapdh* as the positive control. The primers for these genes were previously described [30], [34].

Primers for *Gapdh*, forward: AACTTTGGCATTGTGGAAGG, reverse: GGAGACAACCTGGTCCTCAG, yield a 351 bp product. For *Tnf-α*, forward: CCACCACGCTCTTCTGTCTAC, reverse: CCTTGAAGAGAACCTGGGAGT, yield a 303 bp product. For *Tlr2*, forward: GAGCGAGCTGGGTAAAGTAGAAA, reverse: AGCCGAGGCAAGAACAAAGA, yield a 528 bp product. PCR was performed using *Taq* DNA polymerase (New England Biolabs) with the following amplification conditions: denaturation at 94°C for 2 min; followed by 31 cycles of 94°C for 30 sec, 56°C for 40 sec, 72°C for 50 sec; then extension at 72°C for 5 min. PCR products were subjected to 1.5% agarose gel electrophoresis. To evaluate relative gene transcription levels, a semi-quantitative method was used: Image J software (NIH) was used to collect the gray intensity values of the *Tnf-α* and *Tlr2* PCR bands, which were corrected by the coefficient of *Gapdh* gene expression of the same sample. Student *t* test was used to analyze differences between relative gray intensity of PCR bands. The method has been reported previously [30].

### Immunostaining for TNF-α and TLR2

Middle ear paraffin sections were deparaffinized by immersion in xylene twice, 5 min each; rehydrated in sequential aliquots of 100%, 95%, 80%, and 70% ethanol, 5 min each; and finally, washed in water, 3 min. Then sections were covered with a trypsin working solution (containing 0.1% calcium chloride [pH 7.8]) and incubated 20 min at 37°C in a humidified incubator. Sections were cooled and washed twice with PBS, 5 min each. Then 0.2% Triton X-100 was added to sections for 5 min, followed by 2 more washes in PBS, 5 min each. Next, blocking solution (3% goat serum and 2% BSA) was applied for 1 hour followed by primary antibodies anti-TNF-α (1∶200 dilution) (Abcam, catalog #ab9739) and anti-TLR2 (1∶400 dilution)(Abcam, catalog #ab24192) incubated overnight at 4°C. The next day, sections were washed twice, 5 min each, in PBS; secondary antibody conjugated to Alexa Fluor 488 (1∶500 dilution; Invitrogen) was applied at room temperature for 1 hr. Sections were washed twice with PBS; propidium iodide (10 µg/ml, Invitrogen, catalog #P1304MP) was applied at room temperature for 15 min to counterstain the nuclei. Then sections were mounted in VECTASHIELD® Mounting Medium (Vector Laboratories, Inc.) and observed under an immunofluorescence microscope (Leica DFC500). Images were acquired at 5 to 63× final magnification.

## Results

### Gross examination of craniofacial structures and ears

**Figure 1:**
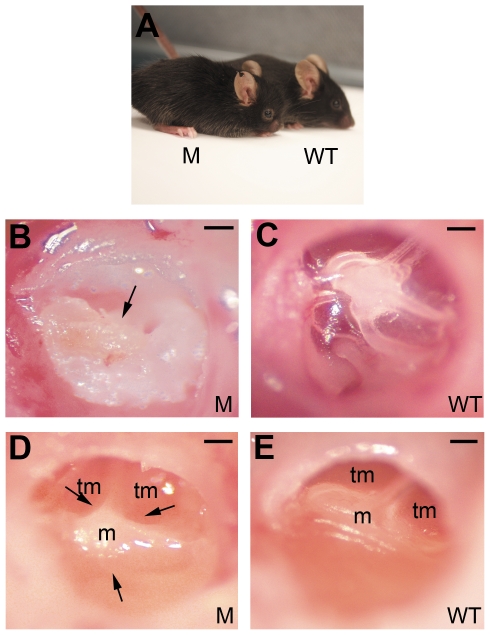
Gross phenotype in *Sh3pxd2b^nee^* mutant mice. **A,** Comparison of the lateral view of a 20-day-old male mutant mouse (left) and a wild-type littermate control mouse (right). The mutant mouse exhibits a domed and rounded skull, and a shortened nose. **B,C,** Macroscopic views of the tympanic membranes of an 11-day-old male mutant mouse (**B**) and a wild-type littermate male control mouse (**C**). The surface of the tympanic membrane is covered by thick, white exudate (arrow) in the mutant mouse ear, making the tympanic membrane adiaphanous and uneven (**B**). In the ear of the control mouse, the ossicles are clearly visible through the smooth, transparent tympanic membrane (**C**). **D,E,** Macroscopic views of the tympanic membranes of a 14-day-old male mutant mouse (**D**) and a wild-type littermate control mouse (**E**). The mutant mouse exhibits a relatively more protuberant malleus, which indicates that there is tympanic membrane retraction (**D**, arrow). The control mouse has a relatively even malleus and tympanic membrane, when compared to the mutant mouse (**E**). (M = mutant, WT = wild-type, m = malleus, tm = tympanic membrane). Scale bar, 200 µm.

At 2.5 months of age, homozygous mutant mice exhibited obvious craniofacial dysmorphology (n = 4, each phenotype, **Figure 1A**) compared to wild-type controls. By anatomical microscopy (Leica S6D), OM was detected in mice as young as 11 days old (**Figure 1B**). The mutant mice showed severe middle ear effusion, sometimes accompanied by tympanic membrane retraction (**Figure 1D**) resembling human OM pathology [35], [36]. OM was detected in all mutant mice over 11 days of age, in contrast to the wild-type control mice.

### Histological observation of middle ears

**Figure 2:**
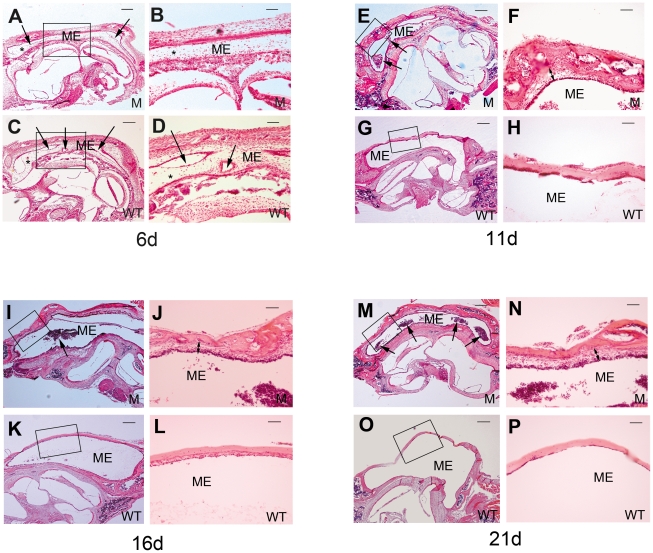
H&E histology shows development of middle ear structures and pathology. Each group of four panels consists of representative images of the middle ears at a single time point after birth: day 6 (**A–D**), day 11 (**E–H**), day 16 (**I–L**), and day 21 (**M–P**). For each time point, the top two panels show images from a *Sh3pxd2b^nee^* mutant (M) mouse (5× magnification, left panel; 20× magnification of boxed region of interest, right panel) and the lower two panels show corresponding images from a wild-type (WT) littermate control mouse. In 6-day-old mice (n = 4, each genotype), the middle ear cavity (ME) is filled with mesenchymal cells (asterisks, **A–D**), and middle ear cavitation has already begun. Cavitation of the middle ear is larger and proceeds earlier in control mice than in mutants (arrows, **A–D**). At 11 days of age (n = 4, each genotype), the mesenchymal cells have disappeared and middle ear cavitation is almost complete in both the mutant (**E**) and control (**G**) mice. Signs of infiltration by inflammatory cells are beginning to show in the middle ears of the mutant mice (**E**, arrows). The middle ear epithelium has also become thickened in the mutant mice (**F**, two-ended arrow). At age 16 days (n = 4, each genotype), more inflammatory cells can be found in the middle ear cavity (**I**, arrow), and the epithelium has thickened even more, as compared to the mice at age 11 days (**J**, two-ended arrow). At age 21 days (n = 4 each genotype), inflammatory cells have significantly increased and are observed throughout the middle ears of the mutant mice (**M**, arrow). The middle ear epithelium has also greatly thickened in the mutant mice (**N**, two-ended arrow).

**Figure 3:**
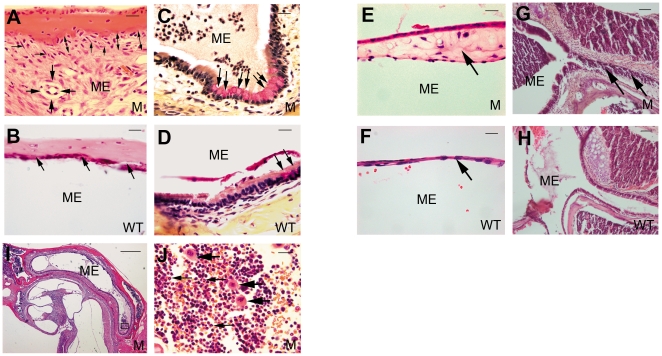
Signs of inflammation and otitis media in *Sh3pxd2b^nee^* mutant mice (M) compared with controls (WT). **A,B,** Representative images comparing pathological changes in the middle ears between *Sh3pxd2b^nee^* mutant mice (**A**) and wild-type littermate control mice (**B**) at the age of 2.5 months. The *Sh3pxd2b^nee^* mutant mice show thickened middle ear mucosae (**A,** two-ended arrow). The middle ear cavity of mutant mice is filled with fibroblastic tissue, showing fibroblastic proliferation (**A,** thin arrows) and capillary hyperplasia (**A,** bold arrow). The wild-type control mice show a thin mucosa (**B,** arrow) and a clear middle ear cavity without fibroblastic proliferation or capillary hyperplasia. **C,D,** Representative images to show goblet cells in the middle ear epithelium in the mutant mice (**C**) and wild-type littermate control mice (**D**) at the age of 21 days. There was a relatively higher density of goblet cells in the middle ear epithelium in the mutant mice as compared to the littermate controls (**C,D** arrows). **E,F,** Representative images comparing tympanic membrane thickness in *Sh3pxd2b^nee^* mutant mice (**E**) and wild-type littermate control mice (**F**) at the age of 2.5 months. The tympanic membrane has become significantly thickened in the mutant mice (**E**, arrow); whereas, the control mice show a thin tympanic membrane (**F**, arrow). **G,H,** Representative images of the eustachian tube in mutant mice (**G**) and littermate control mice (**H**) at age 21 days. The eustachian tube is filled with inflammatory cells in mutant mice (**G**, arrow), compared with normal clearance of the eustachian tube in control mice (**H**). **I,J,** Infiltrated cells are evident in the middle ear cavity of a 21-day-old mutant mouse. **J** is an enlarged view of the boxed region of interest in panel **I**. Infiltrated cells exhibit a polymorphic nucleus (**J**, small arrows). A few macrophages can also be found (**J**, bold arrows). Scale bar(s), panel **I**, 100 µm; panels **G,H,** 50 µm; all other panels, 10 µm. Panel **I,** 5× magnification; other panels, 63× magnification. Panels **C,D,** Mayer's mucicarmine stain; all other panels, H&E stain. ME = middle ear cavity, M = mutant, WT = wild-type control.

Middle ear pathology was assessed at various stages to track OM progression. In 6-day-old mice, middle ear cavitation had already begun both in the *Sh3pxd2b^nee^* mutant mice and wild-type littermate control mice. Cavitation of the middle ear was larger and appeared to proceed earlier in control mice than in mutants (**Figure 2A–D**), but by 11 days, relatively normal development was accompanied by effusion in the middle ear cavities of mutant mice only. Furthermore, these middle ears also showed inflammatory cell infiltration and thickened epithelia (**Figure 2E–H**). In 16-day-old mice, epithelial thickening continued and inflammatory cell numbers increased in the middle ears of mutant mice (**Figure 2I–L**); the trend continued in 21-day-old mutant mice. At 21 days, inflammatory cells had spread throughout the middle ear cavities of mutant mice as OM progressed and became more severe with age. Control mice at 21 days still showed no OM pathology (**Figure 2O,P**). At 2.5 months in mutant mice, the previous middle ear pathology was accompanied by fibroblastic proliferation and capillary hyperplasia (**Figure 3A,B**), a higher density of goblet cells in the middle ear mucosa revealed by Mayer's mucicarmine staining (**Figure 3C,D**), and a thickened tympanic membrane (**Figure 3E,F**), all in contrast to control mice. Histological sections revealed that the eustachian tube in mutant mice was heavily infiltrated by inflammatory cells (**Figure 3G,H**). Individual mutant mice showed differing degrees of middle ear inflammation, at various ages, with excessive effusion and inflammatory cells, most of which were neutrophilic granulocytes, along with a few macrophages (**Figure 3I,J**). Thus, the mucus-secreting ability of the middle ear epithelium was enhanced in the mutant mice, and the excretion ability of the eustachian tube was diminished, leading to excessive effusion in OM. To measure the degree of pathology in OM, five indices were used for semi-quantitative evaluation as shown in **Table 1**. These data revealed that all of the tested *Sh3pxd2b^nee^* mutant mice had signs of inflammation in their ears and that the degree of inflammation in ears of *Sh3pxd2b^nee^* mutant mice was significantly greater than that in ears of wild-type control mice.

**Table 1:** Semi-quantitative evaluation of the outcome of the middle ear inflammation in *Sh3pxd2b^nee^* mice.

Mouse ID	Genotype	Middle Ear Effusion	Inflammatory Cells	Tissue Proliferation	Goblet Cells	Inner Ear Neutrophil Infiltration
1	*WT*	−	−	−	−	−
2	*WT*	+		−	−	−
3	*WT*	−	−	−	−	−
4	*WT*	−	−	−	−	−
5	*WT*	−	−	−	−	−
6	*WT*	−	−	−	−	−
7	*WT*	−	−	−	−	−
8	*WT*	−	−	+	−	−
9	*WT*	+	−	−	+	−
10	*WT*	−	−	−	−	−
11	*WT*	−	−	−	−	−
12	*WT*	−	−	−	−	−
13	*Sh3pxd2b^nee^*	+	+	+	+	−
14	*Sh3pxd2b^nee^*	++	++	+	+	−
15	*Sh3pxd2b^nee^*	+	−	+	−	+
16	*Sh3pxd2b^nee^*	+	++	+++	++	−
17	*Sh3pxd2b^nee^*	++	++	+	−	−
18	*Sh3pxd2b^nee^*	++	+++	+	+	−
19	*Sh3pxd2b^nee^*	++	++	++	+	−
20	*Sh3pxd2b^nee^*	++	++	++	++	−
21	*Sh3pxd2b^nee^*	+	+	+	+	−
22	*Sh3pxd2b^nee^*	+	++	−	+	−
23	*Sh3pxd2b^nee^*	+	++	++	+	+
24	*Sh3pxd2b^nee^*	+++	+++	+	+++	−
25	*Sh3pxd2b^nee^*	+	+	+	+	−

To show the degree of pathological alteration in the middle ears of control and mutant mice, symbols (−, +, ++, and +++) were used with (−) indicating no pathology and (+) to (+++) indicating little to great degree of pathology, respectively. Criteria for pathology included middle ear effusion, density of inflammatory cells, degree of tissue proliferation, density of goblet cells, and degree of inner ear effusion. Both ears were examined, and the recorded finding pertains to the ear with the greatest degree of pathology. We scored 1 point for each +; for a total maximum possible score of 15 points per mouse. The total scored rate (77/195) of 13 *Sh3pxd2b^nee^* mutant mice was significantly higher than that (4/180) of 12 wild-type mice (p<0.01, Chi-square test). Mice from 60–90 days of age were used.

### Scanning electronic microscopy observation

**Figure 4:**
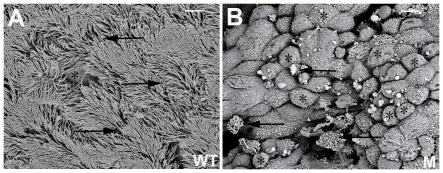
Scanning electron microscopic view of the middle ear mucosa. **A,** Representative image from a 21-day-old wild-type littermate control mouse. Arrows indicate cilia in the mucociliary epithelium of the middle ear with a visually normal morphology. **B** Representative image from a 21-day-old *Sh3pxd2b^nee^* mutant mouse. Arrows indicate the sparsely distributed cilia in the mucociliary epithelium of the middle ear. Cilia also showed abnormal morphology compared with those in the wild-type control. Asterisks indicate the increased numbers of goblet cells in the swollen middle ear mucosa; whereas, only a few goblet cells (**A,** asterisk) could be found in the middle ear mucosa of wild-type control mice. WT = wild-type, M = mutant, Scale bars = 5 µm.

We assessed changes in cilia and goblet cells of the middle ear mucosa from 21-day-old *Sh3pxd2b^nee^* mutant mice using scanning electronic microscopy. Wild-type littermate control mice displayed a thick lawn of morphologically normal, evenly distributed cilia in the mucociliary epithelia of the middle ears. In comparison, the cilia were diminished and sparsely distributed in the mucociliary epithelia of middle ears from *Sh3pxd2b^nee^* mutant mice. Furthermore, an elevated density of goblet cells was evident in the mutant middle ear mucosa. A high density of goblet cells was not found in the middle mucosa from the wild-type control mice. (**Figure 4A–B**).

### Craniofacial measurement

**Figure 5:**
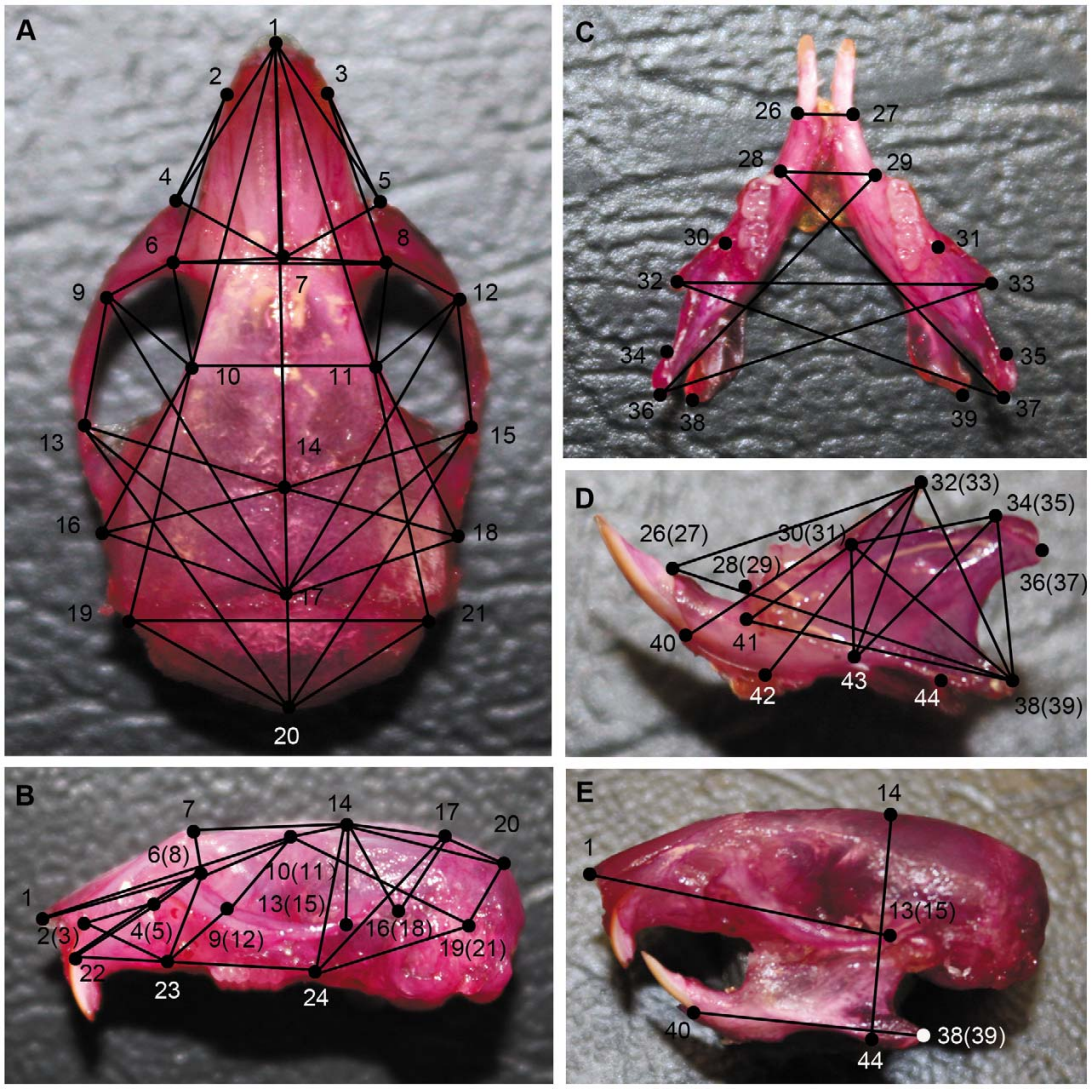
Landmarks and linear distances used in craniofacial measurement*. **A,** Vertical views of skull: 1 Apex point of nose bone; 2,3 Anterior-most point at intersection of premaxilla and nasal bones; 4,5 anterior notch on frontal process lateral to infraorbital fissure; 6,8 intersection of frontal process of maxilla with frontal and lacrimal bones; 7 nasion point; 9,12 intersection of zygomatic process of maxilla with zygoma; 10,11 frontal-squasmosal intersection at temporal crest; 13,15 intersection of zygoma with zygomatic process of temporal; 14 bregma; 16,18 joining of squasmosal body to zygomatic process of squasmosal; 17 intersection of parietal and interparietal bones; 19,21 intersection of parietal, temporal and occipital bones; 20 intersection of interparietal and occipital bones at the midline. (**B**) Lateral view of skull: 22 center of alveolar ridge over maxillary incisor. 23 most inferior point on the premaxilla-maxilla suture; 24 intersection of maxilla and sphenoid on interior alveolar ridge. Other landmarks are identical with the landmarks in panel (**A**). The symmetric overlapping landmarks are in brackets. (**C**) Vertical view of mandible: 26,27 superior-most point on incisor alveolar rim; 28,29 anterior point on molar alveolar rim; 30,31 intersection of molar alveolar rim and base of coronoid process; 32,33 coronoid process; 34,35 anterior-most point on mandibular condyle; 36,37 posterior-most point on mandibular condyle; 38,39 mandibular angle. (**D**) Lateral view of mandible: 40 inferior-most point on incisor alveolar rim; 41 mental foramen; 42 inferior-most point on border of ramus inferior to incisor alveloar. 43 superior-most point on inferior border of mandibular ramus. Other landmarks are identical with the landmarks in panel (**C**), The symmetric overlapping landmarks are in brackets. (**E**) Craniofacial lateral view: 44 the inferior-most point on border of mandibular ramus. Other landmarks are identical with the landmarks in panels (**A–D**), the symmetric overlapping landmarks are in brackets. Straight lines were drawn between the landmarks in panels (**A–E**). In the mutant mice, the linear distance between each landmark pair is significantly shorter than those in the wild-type control mice (Student *t* test, p<0.05), except for the 4 distances: landmark 6-landmark 8, landmark 6-landmark 7, landmark 7-landmark 8, and landmark 10-landmark 11 (Student *t* test, p>0.05). Seven linear distances were selected to evaluate the craniofacial morphology: skull length (Panel **A**, landmark 1-landmark 20), skull width (Panel **A**, landmark 19-landmark 21), skull height (Panel **E**, landmark 14-landmark 44), lower jaw length (Panel **E**, landmark 40-landmark 38(39)), upper jaw length (Panel **E**, landmark 1-landmark 13(15)), nose length (Panel **A**, landmark 1-landmark 7) and interorbital distance (Panel **A**, landmark 6-landmark 8) separately. * Landmarks are mostly following Dr. Joan Richtsmeier's paper on craniofacial dysmorphology in a Down syndrome mouse model [32], and the standard measurement protocol in the Jackson Laboratory Craniofacial mutant source (Jackson Laboratory, Bar Harbor, ME http://craniofacial.jax.org/standard_protocols.html).

**Figure 6:**
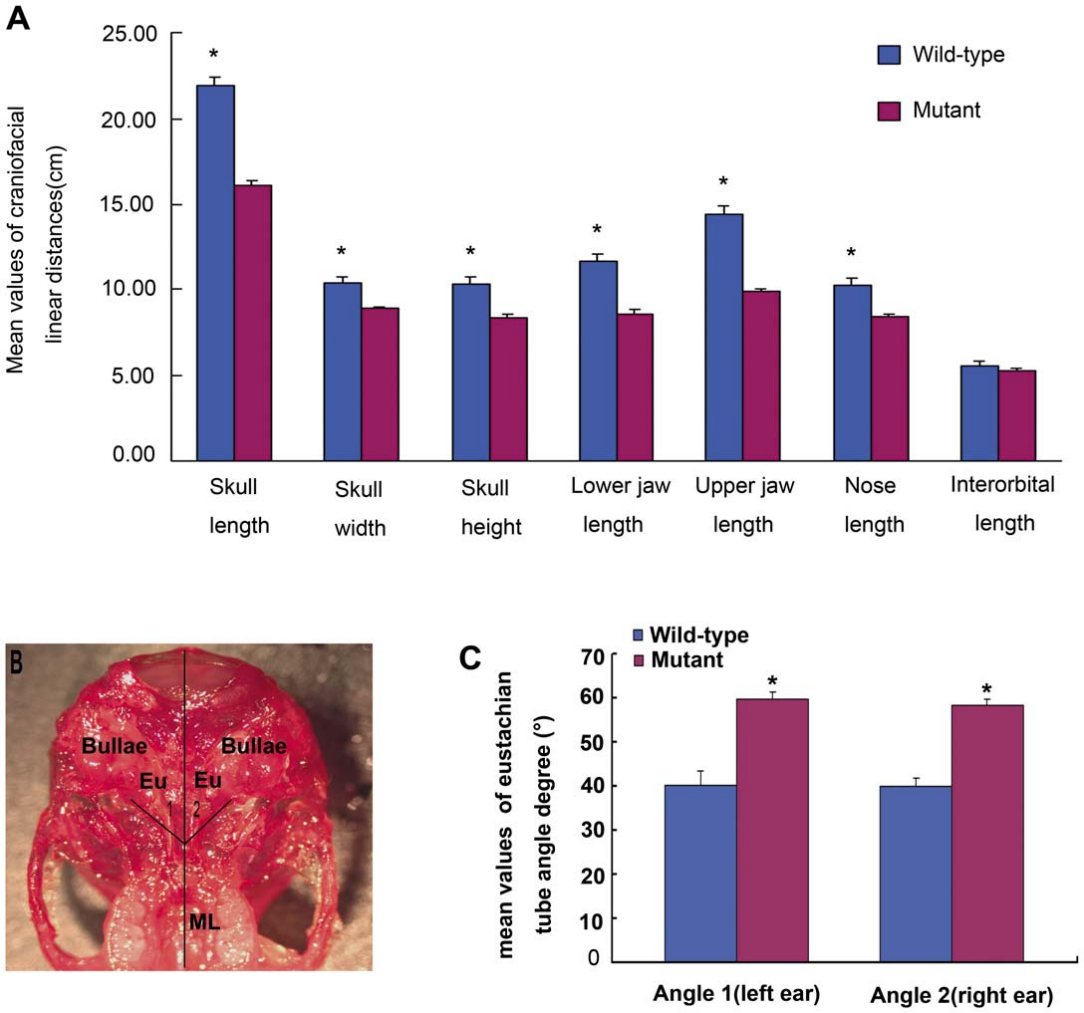
Assessment of craniofacial dysmorphology in *Sh3pxd2b^nee^* mice. **A,** Comparison of linear distance parameters of the craniofacial dimensions of 2.5-month-old *Sh3pxd2b^nee^* (mutant, n = 8, purple bars) and wild-type (control, n = 9, blue bars) mice. All but one measurement parameter yielded statistically significant differences between mutant and control mice (Student *t* test, * P<0.01). The interorbital distance showed no significant difference (Student *t* test, P>0.05). Error bars indicate the standard deviation from the mean for each group. **B,C,** Comparison of eustachian tube angles 1 and 2, from the mid-line of the skull to the left and right ears, respectively. Panel (**B**) shows an antiapical view of a dissected skull with the lines of reference for the measured angles indicated. ML, mid-line; Eu, eustachian tube. Panel (**C**) shows a graphic comparison of the mean values for angles 1 and 2 between mutant (n = 8) and wild-type control (n = 9) mice. The mutant mice had statistically significant larger angles than the wild-type control mice (Student *t* test, *P<0.01). Error bars indicate the standard deviation from the mean of each group.

Craniofacial measurements showed that the mutant mice had a statistically significant (p<0.01) decreased linear distance between craniofacial landmarks for all but four measurements taken. The distances measured have been indicated by black lines in **Figure 5**. The average skull length (**Figure 5A**, landmark 1-landmark 20) of control mice was 22.00 mm; whereas, the average skull length of mutant mice was 16.08 mm. The average skull width (**Figures 5A**, landmark 19-landmark 21) of controls was 10.44 mm versus 8.91 mm for mutants. The average skull height (**Figure 5E**, landmark 14-landmark 44) for controls was 10.34 mm, versus 8.35 mm in mutants. The average lower jaw length (**Figure 5E**, landmark 40-landmark 38(39)) in controls was 11.71 mm, versus 8.63 mm in mutants. The average upper jaw length (**Figure 5E**, landmark 1-landmark 13(15)) in controls was 14.39 mm compared to 9.93 mm in mutants. The average nose length (**Figure 5A**, landmark 1-landmark 7) in controls was 10.28 mm, compared to 8.43 mm in mutants. The average interorbital distance (**Figure 5A**, landmark 6-landmark 8) in controls was 5.60 mm, compared to 5.29 mm in mutant mice. (Measurement data are not shown). All of these measurements showed statistically significant differences in the mean distances between mutant and control mice (p<0.01) except for the interorbital distances (p>0.05)(**Figure 6A**). Between the midline of the skull base and the bony part of the left and right eustachian tubes (angles 1 and 2 in **Figure 6B,C**), mutant mice had significantly larger angles in angle 1: 59.6° versus 40.3° for controls, and in angle 2: 59.64° versus 40.00° for controls (measurement data not shown) (**Figure 6C**, angle 1 and 2; P<0.01). Thus, there were clearly alterations that would produce a more horizontal orientation of the eustachian tubes in *Sh3pxd2b^nee^* mutant mice, paralleling the eustachian tube morphology in humans that may cause a predisposition for OM.

### Time-course ABR thresholds

**Figure 7:**
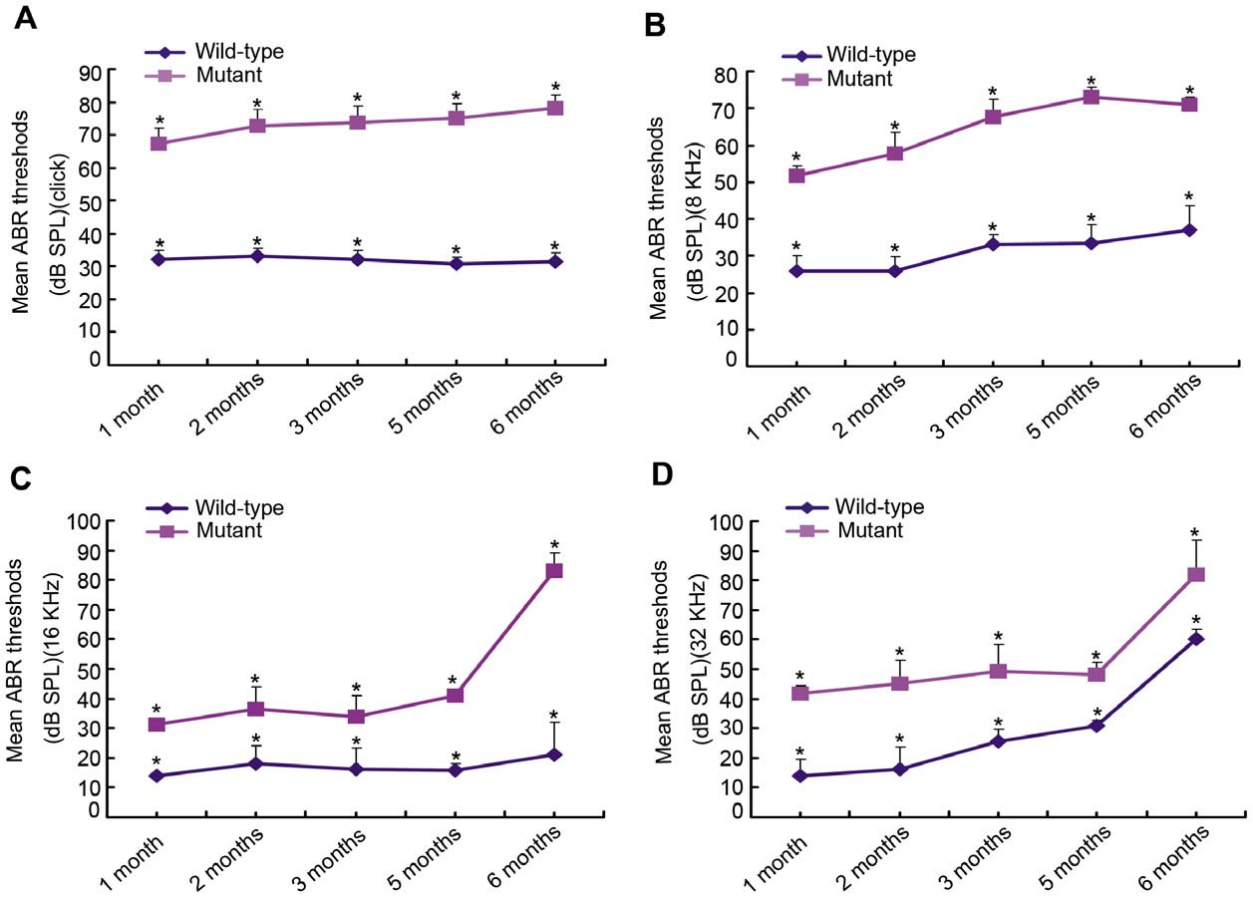
Impaired hearing in *Sh3pxd2b^nee^* mice. A time-course observation of ABR thresholds of right ears from mutant (n = 30) and littermate control (n = 30) mice, tested at the ages of 1, 2, 3, 5, and 6 months. Compared to littermate controls, the mutant mice exhibit significantly higher mean ABR threshold values at every age point and at every stimulus frequency (click stimuli, 8 kHz, 16 kHz, 32 kHz in panels **A, B, C, D,** respectively)(Student *t* test, p<0.01). There appears to be a tendency toward increased hearing threshold at higher stimulus frequencies, both in mutant and control mice. In addition, the higher the frequency, the closer the curves for the mutant and control mice were. This indicates that at the same age point, the gap between the mean ABR thresholds between the mutant and control mice had a declining tendency as the stimulus frequencies increased. Thus, the observed pathology has a greater effect on hearing ability at lower frequencies than at higher frequencies. Error bars represent standard error of the mean.

ABR thresholds were measured in *Sh3pxd2b^nee^* mice and controls several times between one and six months of age and showed a consistent increase in mutant mice (**Figure 7**). ABR threshold mean values above 55 (for click stimuli), 40 (for 8 kHz), 35 (for 16 kHz) and 60 (for 32 kHz) decibel sound pressure levels (dB SPL) indicate hearing impairment [37]. There was a tendency toward elevation at higher stimulus frequencies both in mutant and control mice (**Figure 7C,D**; 16 kHz and 32 kHz). At each time point, the gap between ABR threshold mean values of mutant and control mice tended to decline as stimulus frequency was elevated (**Figure 7B,C**; 8 kHz and 16 kHz). Then we compared the normalized hearing level values from the mutant mice (each mutant hearing levels minus mean hearing level of wild-type), which represent the degree of hearing impairment, between the lower stimulus frequencies (8 kHz **Figure 7B**) and higher stimulus frequencies (32 kHz, **Figure 7D**). We found statistically significant differences at each time point (Student t test, P<0.005, 1 through 6 months). These results demonstrated that the mutation affects hearing at lower stimulus frequencies more than at higher stimulus frequencies.

### Increased mRNA and protein expression for *Tnf-α* and *Tlr2*

**Figure 8:**
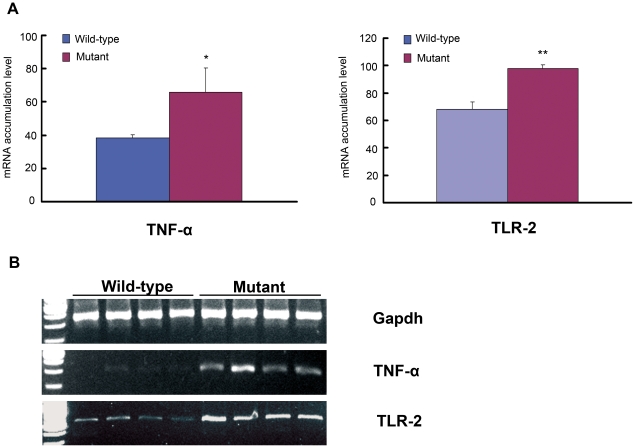
Evidence of active inflammation in ear tissue of *Sh3pxd2b^nee^* mutants up-regulation of pro-inflammatory molecules. Gene expression levels were detected in bullae from 21-day-old *Sh3pxd2b^nee^* mutant mice (n = 4) and wild-type littermate control mice (n = 4). Panel **A** shows in graphic form the semi-quantitative difference in *Tnf-α* (left panel) and *Tlr-2* (right panel) mRNA expression between wild-type and mutant mice as measured relative to *Gapdh* expression. The mRNA accumulation levels of *Tnf-α* and *Tlr2* were significantly higher in *Sh3pxd2b^nee^* mutant mice than in wild-type littermate control mice (Student *t* test, * P<0.05, **P<0.01). Error bars indicate the standard deviations from the means in each group. Panel (**B**) presents the semi-quantitative RT-PCR results summarized in **A**. After 1.5% gel electrophoresis, Image J software (NIH) was used to collect the gray intensity values of each band on the gel. The mRNA accumulation level of each was corrected by the coefficient of *Gapdh* gene mRNA accumulation level of the same sample.

*Gapdh*-correlated, semi-quantitative RT-PCR analysis revealed that mRNA expression levels in the middle ear in four 24-day-old *Sh3pxd2b^nee^* mutant mice were increased for the pro-inflammatory cytokine *Tnf-α* and for cell surface receptor *Tlr2* which aids in pathogen recognition (**Figure 8**, * P<0.05, **P<0.01, respectively).

**Figure 9:**
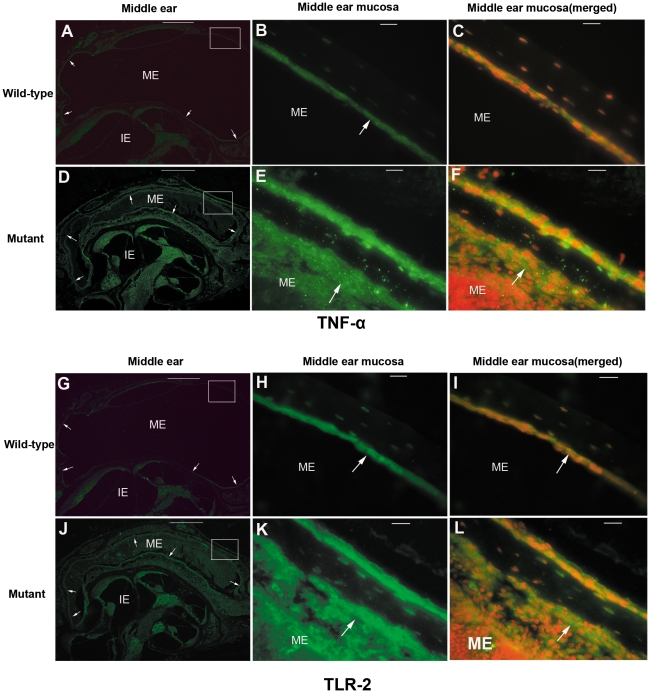
Pro-inflammatory proteins detected in middle ears of *Sh3pxd2b^nee^* mutant mice. Representative immunohistochemistry figures show the staining of middle ear tissue with anti-TNF-α (**A–F**) and anti-TLR-2 (**G–L**) antibodies, revealed with Alexa-Fluor 488 (green). The top 3 panels in each set (**A,B,C** and **G,H,I**) are from wild-type control mice, and the lower 3 panels (**D,E,F** and **G,K,L**) are from *Sh3pxd2b^nee^* mutant mice, all at 21 days of age. Panels on the left (**A,D** and **G,J**) are low magnification images (scale bar = 100 µm) revealing the gross morphology of the middle ears. Middle panels (**B,E** and **H,K**) are enlarged images of the regions of interest boxed in the corresponding left panels. These images reveal the middle ear mucosa and epithelium. Panels on the right (**C,F** and **I,L**) are merged from the middle panels (**B,E** and **H,K**) and images counterstained with propidium iodide in red (not shown separately) to reveal the nuclei in these tissues. ME = middle ear cavity, IE = inner ear, arrows indicate the middle ear mucosa in each panel. In the middle and right panels, scale bar = 10 µm. The staining intensities for both TNF-α and TLR-2 are stronger in the *Sh3pxd2b^nee^* mutant mice than in the wild-type control mice (left panels) Upon higher magnification, it can be appreciated that the staining intensity of both antibodies was much stronger in the middle ear mucosa (middle panels) and localized to the cytoplasm of middle ear epithelial cells (right panels) of the *Sh3pxd2b^nee^* mutant mice, compared with the weaker staining in wild-type control mice. The inflammatory cells in the middle ear cavities of the *Sh3pxd2b^nee^* mutant mice also show a strong cytoplasm staining intensity of both antibodies (**E,F,K,L**).

To determine whether protein levels of TNF-α and TLR2 were upregulated in *Sh3pxd2b^nee^* mice, we analyzed the expression of each in situ. Paraffin sections of middle ears from control and *Sh3pxd2b^nee^* mutant 21-day-old mice were stained with anti-TNF-α and anti-TLR2. In the thickened epithelial walls of the middle ear cavity, TNF-α was upregulated in mutant mice compared to wild-type controls (**Figure 9A–F**). TLR2 immunostaining showed a very similar pattern to that seen for TNF-α (**Figure 9G–L**). In both cases, antibodies specific for TNF-α or TLR2 stained the middle ear mucosa of mutant mice much more brightly than the control samples and this staining was localized to the cytoplasm of epithelial cells and of inflammatory cells. The protein levels seen by immunohistochemistry correlated with the up-regulation of mRNA observed by RT-PCR.

## Discussion

In our study, we explored the hypothesis that gene mutation leading to craniofacial dysmorphology increases susceptibility to OM with effusion and we validated the *Sh3pxd2b^nee^* mutant mouse as an animal model for this. Craniofacial dysmorphology is heritable through gene mutation [37], but how mutations in genes linked to craniofacial development lead to pathologies such as OM is unclear [37], [38]. Using the *Sh3pxd2b^nee^* mouse model, we examined the role of *Sh3pxd2b* mutation in craniofacial dysmorphology and development of OM. Previous studies confirmed that mutation in *Sh3pxd2b* leads to craniofacial dysmorphology and OM [24]; our study examined OM progression and included hearing assessment throughout development. Together with measurement of middle ear inflammatory factor expression, our study more fully characterizes the OM and associated pathologies resulting from *Sh3pxd2b* mutation.

We compared craniofacial structural dimensions of control and mutant mice, analyzed middle and inner ear pathologies, and tested hearing thresholds through various developmental stages. We measured mRNA and protein expression of TNF-α and TLR2, previously reported in OM research [30], [34], [39], [40], [41], to determine whether these inflammatory factors were upregulated in mutant mice that developed inflammation of the middle ear. Our findings showed that all *Sh3pxd2b^nee^* mice were born with craniofacial dysmorphologies and developed OM, as early as 11 days of age, with associated pathologies progressively worsening. Furthermore, the mutant mice showed impaired hearing that was statistically more pronounced at lower frequencies. The type of hearing impairment caused by otitis media is conductive hearing loss when active otitis media exists. It is difficult to perform bone-conduction ABR in the mice because the mouse head is so small. In human patients with active otitis media, lower frequencies are more compromised than higher ones [42]. The mutant mice showed increased mRNA and protein levels of TNF-α and TLR2, which are consistent with the activation of inflammation pathways shared with other OM in mice and humans [29], [37], [43].

Agreeing with a previous study [24], our findings confirmed craniofacial dysmorphology in *Sh3pxd2b^nee^* mutant mice and, for the first time in this model, showed eustachian tube dysmorphology, which has been closely linked to OM development in children [1], [2]. Specifically, craniofacial dysmorphology led to a positional change of the eustachian tube. The angle between the midline of the skull base and the bony part of the eustachian tube was increased in mutant mice, on both the right and left sides, thus imparting a more horizontal orientation to the bony part of the eustachian tube. Other signs of pathology in the mutant mouse eustachian tube included thickened epithelium, presence of inflammatory cells, and a narrowed tympanic cavity opening of the eustachian tube. This is the first demonstration in mouse of these alterations of the eustachian tube which are associated with craniofacial dysmorphology and are all phenotypic outcomes that accompany OM.

Time-course examination of OM pathology progression revealed 11-day-old mutant mice with a much higher degree of middle ear effusion (a primary sign of OM) than control mice. By 2.5 months, mutant mice also showed fibroblast proliferation, and increased goblet cells and inflammatory cells. These phenotypic outcomes are consistent with findings in human patients [44], [45]. Older mice also exhibited more tissue repair reaction in the middle ears.

Cilia present on the mucociliary epithelia of the healthy middle ear are critical for the clearance of secretions. Goblet cells associated in normal numbers with the mucociliary epithelia of the middle ear mucosa are involved in the production of mucosal secretions and contribute to a normal immune response [38], [46], [47]. Our findings reveal morphological abnormalities of cilia in the mucociliary epithelia of middle ears that may cause an inability to clear secretions in *Sh3pxd2b^nee^* mutant mice. We also found a high density of goblet cells in the middle ear mucosa of mutant mice, which may further explain the accumulation of excessive exudate in the middle ear cavities of these mice.

Mutant mice demonstrated impaired hearing upon auditory brainstem response testing, which determines the minimum threshold of hearing at different sound frequencies. We examined hearing at ages 1, 2, 3, 5 and 6 months and found significantly impaired hearing as early as 1 month. A tendency toward increased hearing threshold with age was evident at higher frequencies. Overall, however, hearing impairment of mutant mice was more pronounced at lower frequencies. Thus, the pathological hearing effects associated with this mutation are complex because hearing is affected differently depending on sound frequency and mouse age.

When craniofacial dysmorphology is present, secretions cannot be cleared from the middle ear cavity through the eustachian tube, and bacteria can accumulate, leading to inflammation [38]. Failure to clear secretions because of eustachian tube dysfunction can probably explain the upregulation of inflammatory factors in *Sh3pxd2b^nee^* mice. Toll-like receptors (TLRs) function on host cell surfaces to recognize pathogens and recruit cytokines and other immune effectors. Other studies have shown that TLR2 was present in the middle ears of patients with OM [34], [39], [40], [41]. Our findings show that TLR2 was upregulated in the mutant mice.

### Conclusion

Our study shows that a mutation in the *Sh3pxd2b* gene, which affects craniofacial development in mutant mice, led to OM that mimics the human disease in a number of important physiologic and metabolic parameters. This is the first mouse model to exhibit eustachian tube morphology that mimics the eustachian tube morphology in humans which has been linked to OM. Further examination of genes such as the *Sh3pxd2b* gene are warranted to further elucidate how these proteins are involved in onset and progression of craniofacial dysmorphology and OM. Identifying the relationships between *Sh3pxd2b* and other genes and between SH3PXD2B protein and other proteins is an important step toward understanding craniofacial development and the onset of OM, ultimately promoting treatment and prevention options.
